# Women’s participation in the prevention and control of dengue using environmental methods in the global south: a qualitative meta-synthesis

**DOI:** 10.1186/s12939-022-01726-0

**Published:** 2022-09-23

**Authors:** Cathy Mungall-Baldwin

**Affiliations:** 1 Institute of Health and Wellbeing, University of Glasgow, Institute of Health and Wellbeing, University of Glasgow, 1 Lilybank Gardens, Glasgow, G12 8RZ Scotland, UK; 2 School of Public Health and Community Medicine, University of New South Wales, UNSW Medicine, University of New South Wales, Sydney, NSW 2052 Australia

**Keywords:** Dengue, Gender, Qualitative, Meta-synthesis, Women’s role, Global south, Prevention, Vector control

## Abstract

**Background:**

Dengue, a mosquito-borne viral disease, causes significant mortality and morbidity in low- to middle-income countries. A body of research indicates that women can be effective in implementing vector borne disease control, but they still face inequitable opportunities for participation, leadership and decision-making in the execution of dengue prevention and vector control programmes. Yet implementing informal environmental management practices to prevent mosquito vector breeding forms part of their domestic household responsibilities. Understanding the enablers and barriers to women’s equitable roles with men in formal and informal disease prevention, and the benefits of their participation could help to increase their role and may be a contributing factor to reducing disease rates. The objective of this qualitative meta-synthesis was to synthesise evidence about women’s roles in dengue prevention and control in the global south and generate insights around the barriers, enablers, and benefits.

**Methods:**

Eight databases were searched from inception to 7^th^ December 2020. One investigator independently reviewed all titles and abstracts for relevant articles. Grey literature was searched using 34 websites of global health and international development organisations.

**Results:**

A total of 18 articles representing qualitative research or the qualitative component of mixed methods studies from Latin American and Caribbean (*n* = 8), Asia (*n* = 9), and one international review were included in the meta-synthesis. Relevant scholarship from Africa was lacking. This meta-synthesis revealed five unique themes surrounding women’s participation, seven categories of barriers, six of enablers, four health, well-being and social benefits for individuals, and four for communities .

**Conclusion:**

An analysis of the results confirmed that women’s participation in dengue prevention was not gender equitable, gender sensitive nor transformative although women are the primary human resource for household and community-based prevention. Women demonstrated specific qualities aiding successful implementation. Corrective action is urgently needed to shift unhelpful gender norms, and empower women into leadership and decision-making roles.

## Introduction

There is evidence of gender inequity in the implementation of community-based prevention for dengue, meaning that women do not have fair and just access alongside men. This may be a contributing factor to an increase in outbreaks in low- to middle-income countries (LMICs). Gender equity is defined as ´fairness and justice in the distribution of benefits and responsibilities between women and men (…*who have*…) different needs and power…’ [117]. Involving women in the management and leadership of the prevention of dengue and other mosquito-borne viral diseases (MBVD) using environmental management measures offers a major opportunity to improve dengue control.

Dengue, an acute mosquito-borne arboviral disease, is primarily transmitted to humans by *Aedes aegypti* and *Ae. albopictus* mosquito bites. About 400 million dengue infections occur worldwide annually; it is endemic in 128 countries [13] putting more than 2.05–3.74 billion people at risk [120]. The number of cases reported to the WHO has increased eight-fold in the last two decades [124]. Overall, the male-to-female ratio is 1:1. The gap has decreased in the last two decades where in 1990s, females had higher age-standardised incidence rates (ASIR) [123] of 1.10 and 1:08 in 2019 [123] Global burden of disease reports and disease modelling suggest that dengue is vastly underreported [124] and misdiagnosed [119]. It can be speculated that this may be more so for women who do not report cases or attend hospitals.

Dengue causes significant morbidity and mortality, with severe and debilitating symptoms [31, 94], and in extreme manifestations, dengue haemorrhagic fever (DHF) and dengue shock syndrome [28, 41]. It forces infected people and those caring for family to exit the labour force, with high costs in prevention, control and management for governments [28, 48, 102]. Prevention of transmission is a major public health problem in tropical and sub-tropical regions [95, 119].

Dengue has a variety of environmentally-driven causal factors such as rapid, unplanned urbanisation [28]. Mosquito breeding occurs in environments with inadequate water, sanitation, infrastructure and housing [64] in water-holding vessels and spaces such water-storage containers, solid waste, guttering, underground water sources, tyres, flower pots, discarded containers and many others holding stagnant water [51, 58]. Whilst a vaccine is available in Latin America, it was withdrawn from use in South East Asia and few countries have implemented it due to safety concerns for seronegative vaccine recipients [119]. There is no antiviral therapeutic treatment [66]. Insecticide-treated mosquito nets and larviciding of breeding sites, traditionally carried out by male vector control patrols, meet increased mosquito resistance. They are not practical in the times and places where *Aedes *mosquitoes are most active outdoors at dawn and dusk, although they can bite any time of the day. Typical education for dusk/dawn prevention for malaria mosquito vectors does not significantly protect against dengue [36].

Community-based, participatory, gender equitable mosquito-vector control practices with health promotion and supportive multisectoral government policies across health, environment, education, gender, and urban planning are required for successful dengue control [9, 12, 50, 58, 105, 120]. In 2021, a biological control method of infecting *Ae. aegypti* mosquitoes with a virus-fighting bacteria, wMel, that reduces their ability to spread dengue resulted in a 77% decline in dengue cases and hospitalisations in the intervention areas in Indonesia [107]. It is yet to be formally recognised by WHO, and environmental prevention methods remain critical.

Environment vector control practices include: environmental *management* (destruction of vector habitats, street cleaning, removal of discarded tyres); *modification* (waste systems, piped water, protecting water infrastructure); and *manipulation* (protecting uncovered water sources and containers, i.e. covered/face-down water storage containers); and mosquito-proof housing to prevent biting (protective screens/doors, no open guttering) [9, 12, 50, 58, 59, 62, 67, 94, 120]. Historically, women and health workers in LMICs had limited roles in community leadership and decision-making, and therefore in environmental vector control [42, 101, 110] due to socio-cultural factors e.g. norms, beliefs, perceptions, attitudes, roles, gendered power relations and socio-economic, material and structural factors [6, 19, 25, 32, 39, 45, 63].

### Gender inequity in dengue prevention

Intersectoral frameworks to study the relationship between gender equity and Neglected Tropical Diseases (NTDs) in LMICs have so far been applied to women’s and men’s differential disease rates, access to healthcare and treatment, and caring roles [11, 68, 71, 81, 91, 98]. Most MBVD prevention studies (as a sub-set of NTDs) represent women’s roles as unpaid organisers or health education volunteers or managing household water sources or waste as part of their domestic workloads [23, 31, 46, 84, 96, 115] whilst men mainly hold formal paid roles [﻿^25^, ^32^, ^39^, ^42^, ^45^, ^73^, ^101^, ^110^]. Although women have run small, informal vector control businesses, gaining socio-economic empowerment and inclusion, they encounter economic, procedural, political, and legal obstacles, and lack leadership skills [39]. In Latin America, gender inequity is identified as a possible factor for poor dengue prevention results [110]. In East Africa, gender-based violence (GBV) may be a barrier to equitable prevention [79, 98, 109].

With rising global dengue rates, understanding the barriers to and enablers of women’s increased participation and leadership is essential to knowing how to enhance women’s roles. Women’s participation in community-based interventions for other public health problems evinces positive results, such as a housing improvement programme in urban Guatemala to prevent Chagas disease, where women and men were driven to implement housing improvements to reduce disease vectors by different factors, but both were more likely to take action when collaborating together [104]; the surveillance and implementation of measures to prevent Guinea Worm by female volunteers in Ghana led to a 36% drop in transmission rate [112]; and the Micro-Finance for AIDS and Gender Equity programme in South Africa that engaged women, men and boys around intimate partner violence over two years and afterwards, women participants reported 55% fewer acts of violence [122].

Few studies have explored women’s leadership of urban environmental vector control [25, 32, 45]. By pooling insights from the body of qualitative studies on dengue control, this study will contribute a more in-depth understanding of what is already known, and emergent key themes and insights that may also apply to other MBVDs (cf. [73]).

#### What data is there?

More research is needed on gender equity in vector control interventions and the NTD workforce [68, 110] and none have confirmed the existence of gender equitable large-scale vector control programmes [25]. Whilst social science research suggests how women can contribute to dengue prevention [73], a recent mixed methods systematic review reported that most vector-borne disease studies lack detail on women’s roles [39]. This study only briefly identified some general barriers to women’s participation, but not specific to dengue prevention. A preliminary literature review for the present article found no systematic inquiry into this topic or interventions that have trialled ways of removing the barriers to women’s leadership and delivery roles in environmental prevention on a multilevel societal basis, i.e. micro>meso>macro>meso>micro.

A sizeable body of quantitative work documenting the results of Knowledge, Attitudes and Practice (KAPs) surveys from Latin America and Asia (e.g., [31, 34, 53, 108]) offers insights into communities’ understandings of dengue and preventative actions. Limited qualitative components show how understandings are formed and the results expected. Fewer drill down to how gender intersects with dengue prevention and control measures. Most disaggregate survey participant data by biological sex or a binary construction of gender, a socially constructed concept that varies over time and is influenced by context [82], and the socio-culturally-influenced roles occupied by women in vector control programmes. Women’s role is an under-scrutinised topic with most studies concentrating on malaria (e.g., [25, 86, 101]).

#### This meta-synthesis

This study emphasises depth of data rather than of scale, hence the selection of a qualitative meta-synthesis method. The community-level is defined as ‘a unit broader than the individual woman’s household and narrower than national, state or province level interventions. The geography and scope of the community may vary.’ ([39], p.3). The definition of ‘community’ remains broad for the purpose of the wide range of socio-cultural contexts of the identified studies.

The research questions were: 1) What activities do women participate in to prevent and control dengue in their neighbourhoods? 2) What are the enablers of, and barriers to women’s participation on an equitable basis with men? 3) What are the health, well-being and social benefits of women’s participation?

## Methods and analyses

This study used a meta-synthesis approach and drew from qualitative synthesis methods developed by Butler et al. [15], Thomas and Harden [100] and Noblit and Hare [75]. It is reported using a modified version of the *Enhancing transparency in reporting the synthesis of qualitative research* (ENTREQ) statement [103].

Meta-ethnography techniques ([35], p.2) were used to synthesise and interpret evidence from multiple studies to develop a meta-theory or understanding by identifying and integrating explanatory concepts and themes from various contexts, and grouping and synthesising those that form a coherent explanatory body. The purpose is interpretation not prediction unlike a quantitative review ([100], p.3), [26], p.326), so not every published study is included if fewer contain the same range of concepts and themes ([100], p.3). Meta-narrative techniques were adopted to give the resulting synthesis a coherent, linear structure.

### Search methods for identification of studies

Butler et al.’s [15] three-pronged search strategy was used and the following databases searched between 19th June 2019 and 7th December 2020: Medline/PubMed, PsycINFO, ProQuest, Scopus, EMBASE, Web of Science; EBSCOhost-Medline and Psychology & Behavioural Sciences Collection and free text searching of Google Scholar and library catalogues at the Universities of Glasgow and New South Wales (UNSW). At the suggestion of two anonymous peer reviewers, two additional databases, Lilacs and Scielo were searched, but these generated no new titles in English, only several duplicates. Keywords on women, gender and dengue prevention and control strategies (listed in Table 3 Appendix) based on the author’s knowledge were trialled in Medline/PubMed. Gunn et al.’s [39] list of websites of organisations working on international development, gender, global health and MBVD prevention was used for a free-text search for ‘grey literature’ reports on dengue – see Tables 3 and 5 in the Appendix).

### Selection of studies

The inclusion criteria were: 1) studies discussing women’s participation (separately or with men) in formal or informal prevention and control interventions, and/or health education and promotion; 2) written in English; 3) that included empirical data and/or theoretical exegesis based on interpretation of primary or secondary empirical data; and 4) addressed local socio-residential contexts in LMICs. Empirical ‘data’ were defined as ‘first order’: informants’ quotes; and ‘second order’: author interpretation and exegesis ([15], p.245). Exclusion criteria were papers lacking a qualitative analytical component ([88], p.3), and sociological or anthropological explanation of gendered dynamics, and studies of chemical (i.e. fogging and spraying) or biological control (i.e. placing guppy fish or *Bacillus thuringiensis israelensis (Bti),* a biological larvicide, inside water tanks). Mixed methods studies were included if they contained qualitative or ‘line of argument’ data (see results Table 4 in Appendix) The search was limited to the first 100 titles whose title, abstract or text contained the words (dengue) AND (gender or women or female) AND (prevention and/or control) and the abstract and content scanned as the purpose is interpretation not quantification. A purposive sampling approach [26] was used to scan the abstracts, shortlist and then select articles.

The review was carried out by a single author with the potential for selection bias to undermine and limit the study’s rigour, but mitigated by a careful quality appraisal strategy, although a team review is not a requirement of qualitative reviews. After duplicate titles were removed, papers were screened more closely in random order, and data extraction performed, reporting the following information: 1) Number of entry; 2) authors; 3) publication year; 4) journal; 5) country setting/context; 6) research questions or aims; 7) theoretical/philosophical perspective/rationale; 8) study population; 9) sample size; 10) methodology; 11) data collection method; 12) analysis method; and 13) results and relevant insights ([4], p.3; [100], p. 4).

### Quality appraisal

Each of the short-listed studies was appraised using the 10-item Joanna Briggs Institute (JBI) Critical Appraisal Checklist for Qualitative Research) [61]. Item 6 was adapted from cultural or theoretical locatedness of the authors to acknowledgement of the socio-cultural context. Five of the 10 following criteria had to be satisfied to be included in the meta-ethnography: 1) philosophical perspective, 2) research question/objectives, 3) data collection methods, 4) representation and analysis of data, and 5) congruency of interpretation of results with the overall research methodology; and 6) acknowledgement of socio-cultural context and 7) researcher’s influence; 8) appropriateness of methodology for uncovering the study sample’s voices, and their suitable represented; 9) ethical approval; and 10) conclusions linked to the interpretation/analysis of data.

### Meta-analysis and synthesis

I drew from education researchers, Noblit and Hare [75] three ways of relating studies through an iterative process of ‘translation’: comparing concepts, themes and lines of argument between studies and assessing the extent to which they are similar (1. ‘reciprocal synthesis’), different (2. ‘refutational synthesis’) ([93], p. 37) or dissimilar but connected studies as a body of work represent a line of argument made up of various parts (3. ‘line of argument synthesis’) ([35], p.1). I integrated this theory with the three stages of a thematic synthesis described by Thomas and Harden [100] where: 1) textual, empirical data extracted into an additional column marked ‘Findings’ in the Excel sheet were hand-coded line-by-line; 2) ‘descriptive themes’ and concepts were identified and coded; and 3) meta-‘analytical themes’ were generated, and titled.

I performed an inductive analysis to identify and describe the emerging meta-concepts and themes, organise them into an argument showing the connections to interpret the findings ([100], p.3) and answer the meta-synthesis research questions (an ‘interpretive synthesis’ ([24], p.36) not an ‘aggregated synthesis’ summarising and describing the data).

## Results of the meta-synthesis

### Results of screening

**Fig. 1:**
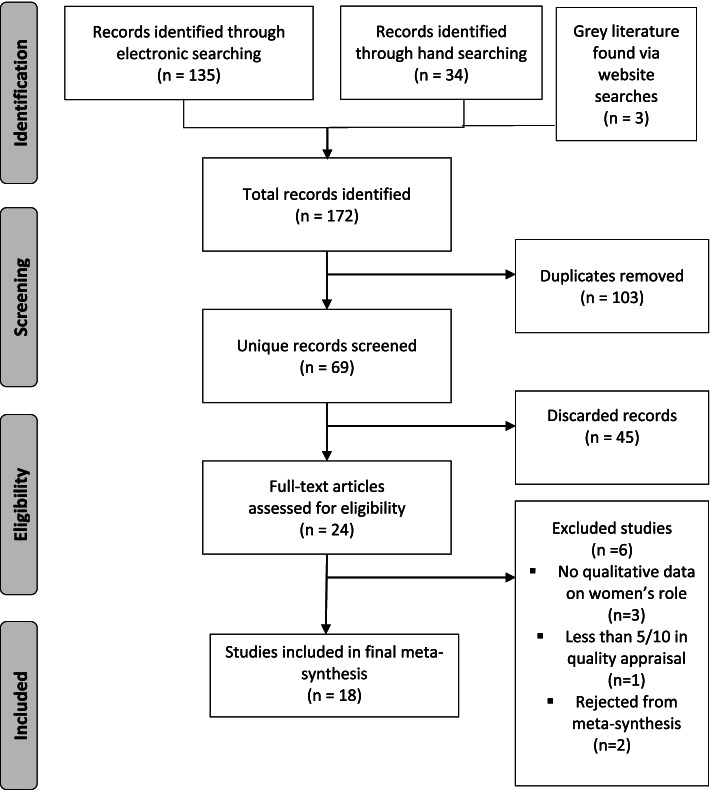
PRISMA flow diagram depicting the study selection process

Sixty-nine unique papers were identified for screening, 24 assessed for eligibility and 18 included. Reasons for the decision to exclude 45 papers from the initial scan are listed in Table 6 in Appendix. The results of the selection process are shown in Fig. 1, *Prisma Flow Diagram*. Three papers were excluded after the appraisal screening which on closer inspection, did not meet the criteria. One paper scored 2/10 during the quality appraisal so was removed. Twenty papers scored 5/10 or more (see Table 1) and were entered into the meta-synthesis.

**Table 1:** Results of the Quality Appraisal (N of papers selected for the meta-analysis = 21)

Author and year of publication	Abeyewickreme et al. [1]	Amani et al. [2]	Arunachalam et al. [5]	Caprara et al. [16]	Echaubard et al. [29]	Espino et al. [33]	García-Betancourt et al. [37]	Idalí-Torres [47]	Lloyd et al. [60]	Mohamud et al. [70]	Nading [73]
**JBI Checklist question**											
1) congruity between the stated philosophical perspective and research methodology?	X	Y	Y	Y	Y	Y	Y	Y	Y	X	Y
2) …the research methodology and research question or objectives?	Y	Y	Y	Y	Y	Y	Y	Y	Y	Y	Y
3) …the research methodology & methods used to collect data?	Y	Y	Y	Y	Y	Y	Y	Y	Y	Y	Y
4) …the research methodology & representation & analysis of data?	X	Y	Y	X	X	X	Y	Y	X	Y	Y
5) …the research methodology & interpretation of results?	Y	Y	Y	Y	Y	Y	Y	Y	Y	Y	Y
6) Is there a statement locating the researcher culturally?	N	N	N	N	N	N	N	Y	N	N	Y
7) Is the influence of the researcher on the research, & vice- versa, addressed?	N	N	N	N	N	N	N	N	N	N	Y
8) Are participants, & their voices, adequately represented?	N	N	X	N	N	N	X	X	N	Y	Y
9) Is the research ethical according to current criteria or for recent studies/evidence of ethical approval by an appropriate body?	Y	N	Y	Y	Y	Y	Y	N	Y	N	Y
10) Do the conclusions drawn in the research report flow from the analysis or interpretation of the data?	Y	Y	Y	Y	Y	Y	Y	Y	Y	X	Y

Author and year of publication	Oliveira & Caprara [78]	Pérez-Guerra et al. [85]	Respati et al. [89]	Stewart Ibarra et al. [96]	Tapia-Conyer et al. [97]	Whiteford [111]	Winch et al. [113]	Winch et al. [114]	Wong et al. [116]	Zuhriyah et al. [125]
**JBI Checklist question**										
1) congruity between the stated philosophical perspective and research methodology?	Y	Y	Y	Y	Y	Y	Y	Y	Y	X
2) …the research methodology and research question or objectives?	Y	Y	Y	Y	X	Y	Y	Y	Y	Y
3) …the research methodology & methods used to collect data?	Y	Y	Y	Y	X	Y	Y	Y	Y	Y
4) …the research methodology & representation & analysis of data?	Y	Y	Y	Y	X	Y	Y	Y	Y	Y
5) …the research methodology & interpretation of results?	Y	Y	Y	Y	X	Y	X	Y	Y	Y
6) Is there a statement locating the researcher culturally?	N	N	N	N	N	N	N	N	N	N
7) Is the influence of the researcher on the research, & vice- versa, addressed?	X	N	N	N	X	N	N	N	X	N
8) Are participants, & their voices, adequately represented?	Y	N	X	Y	X	Y	X	X	Y	Y
9) Is the research ethical according to current criteria or for recent studies/evidence of ethical approval by an appropriate body?	Y	Y	Y	Y	X	N	X	N	Y	N
10) Do the conclusions drawn in the research report flow from the analysis or interpretation of the data?	Y	Y	Y	Y	X	Y	Y	Y	Y	X

Y = yes, N = no, X = not applicable

## Results of the meta-synthesis

### Q1: What activities do women participate in to prevent and control dengue in their neighbourhoods?

#### Women’s participation: themes and concepts

The 18 included papers were sub-divided into urban or peri-urban studies from Latin America and the Caribbean (*N* = 8), and urban, peri-urban or rural studies from South East Asia (*N* = 9), and 1 international review. The results of the synthesis are presented as answers to the three research questions, and grouped according to the key themes and concepts identified.

### Women’s participation: themes and concepts

#### Prevention practices

Home-based women and girls employed a suite of ‘prevention practices’ informally or through formal interventions. Most were duplicated across settings, enabling a *reciprocal synthesis*. Informal household-centred prevention comprised three main strategies:

1. Managing and maintaining clean water such as covering drinking vessels or adding ingredients perceived to purify water supplies [96, 111], as dirty water supplies presented a chief worry in poor communities such as Villa Francisca, Dominican Republic, where women couldn’t afford purified water [111].
2. Domestic waste management [1, 73, 85, 111] with women removing empty containers to prevent collection of stagnant water and mosquito breeding in Carolina, Puerto Rico [85]; and cleaning the street outside the home, such as in Ciudad Sandino, Nicaragua, and Fortaleza, Brazil, due to inadequate municipal waste collection [16, 73].
3. Daily household cleaning and maintenance [1, 73, 96] such as in Sri Lanka’s Gampha district [1], Ciudad Sandino, Nicaragua [73] and Machala, Ecuador ([96], p.9). Practices always reflected a gendered division of labour.

Similar practices were implemented in formal interventions run by regional or local government, non-governmental organizations (NGOs) or unions (e.g. the Lao PDR Women’s Union) [60], on a community-wide scale [1, 16, 33, 60, 113], for example, eliminating empty containers, bottles, tins, bowls or tyres or covering them to prevent rainwater entering, reversing them or covering them when in use [1, 113].

In some interventions, women performed entrepreneurial environmental management activities, such as manufacturing a community supply of 9528 adult mosquito traps from recycled bottles in rural Cambodia [29]. In Celestún, Mexico, *Chen Kole ‘Lob* (“only women” in local language, Yucatec Maya) is a group formed by 17 women to recycle plastics and packaging due to fears about the health risks of coastal waste disposal (Hanson in [2]). Other activities involved strategic planning for vector reduction, e.g., Cambodian women contributing to ‘participatory epidemiology mapping sessions’ to identify mosquito transmission zone boundaries [29].

#### Health education and promotion

Unpaid or poorly paid roles in health education and promotion involved, for example, women going house-to-house to raise dengue awareness in Khyber Pakhtunkhwa, Pakistan [70]; community mobilisation in Fortaleza, Brazil [16]; reminding and supervising householders to inspect and cover water containers in Manilla, Philippines [33]; and advising on mosquito control in Ciudad Sandino, Nicaragua [73].

#### Folk theories

Through intense observation of vector breeding environments, women developed ‘folk’ theories of mosquito elimination and bite prevention (*reciprocal synthesis*), but with variation in beliefs (*refutational synthesis*). Villa Franciscan women (Dominican Republic) ‘cleansed’ their cooking water, fanning the air near water storage containers to prevent mosquito larvae, although knowing that this alone would not prevent disease [113]. Disbelieving standard primary prevention methods, housewives in the Klang Valley, Malaysia covered themselves in lemon grass oil among other measures [116].

#### Women’s responsibility for health, domesticity and dengue prevention

Women’s knowledge of and responsibility for caring for family health alongside the burden of domestic work, and thus domestic dengue prevention, was identified across most papers, enabling a *reciprocal synthesis*. In Villa Francisca, Dominican Republic, the senior women of the household, especially older mothers, had greater knowledge of and responsibility for family health and healthcare. They implemented prevention measures for diseases as well as performing domestic work [113]. In Gampha District, Sri Lanka, women were cultural guiding figures and education providers for children, and implemented dengue prevention by cleaning and managing solid household waste [1]. In Machala, Ecuador, women held responsibility for family health and dengue prevention, a challenge for those who worked: “…we get home late, sometimes tired, and sometimes we don’t have time – even though it doesn’t take much time (PA woman)” ([96], p.8).

Responsibility for family health extended to community-level prevention. In Bandung, Indonesia, women, the primary carers, were obliged to provide environmental care by managing mosquito breeding sites [89]. In Fortaleza, Brazil, female participation was very strong in several arms of a cluster randomised controlled trial (CRTC) due to women’s leadership in household activities, controlling dengue, and now community mobilisation for the CRCT [16]. In Ciudad Sandino, Nicaragua, prevention was seen as a “women’s issue” ([73], p.15) by citizens and the city’s low-paid health workers - *brigadistas*, 90% of whom were female and single mothers or de facto heads of households, although the gendered aspect wasn’t explicit in Nicaraguan or global health policy. Most were reportedly women as they were ‘smarter’, those younger and single having adaptability and close knowledge of households, meaning responsibility for health ([73], p.136).

#### Gendered roles in prevention

The clear gendering of roles in prevention is another theme leading to a *reciprocal synthesis*, with minor blurring of the boundary between gender roles only in Carolina, Puerto Rico [85]). The qualitative component of a CRCT in Chennai, India, showed women with traditional, sharply demarcated gender roles: responsible for family care, domestic work and dengue prevention through collecting piped water-storing and cleaning water containers [5]. Men did occupational work and income-generating activities. In Villa Francisca, Dominican Republic, 77% of women didn’t work outside the home due to traditional gender roles including water management to prevent disease, and lacked opportunities [111]. In Chennai, income generation was not the only reason for men’s absence from prevention. One fisherman husband spent time at the liquor shop with his peers when not at sea [5].

Women and men’s dengue concerns and prevention priorities were often split along gendered lines. In Carolina, Puerto Rico, women were more concerned about infrastructure problems potentially contributing to further transmission, and managed domestic breeding sites whilst men removed tyres and rubbish outdoors [85]. Women in Malang, East Java, Indonesia, reduced risks of bites through community-based mosquito management, whilst men preferred fogging. This was explained by men’s role as providers, despite women’s increased participation in and support of prevention. Men saw prevention as housewives’ responsibility through organised structures such as the Women’s Union, whilst women saw men’s role in managing outdoor environments, e.g. cleaning ditches through voluntary groups. Women were excluded from decision-making in prevention programmes and local leadership, although they possessed knowledge and health education [125].

In Chennai, India, even if a man wished to help, the gendered division of labour and daytime locations formed obstacles: "…we must collect pipe water supplied by metro water department for cooking and drinking. The pipe water is supplied during daytime only, at the time I go to office. So how can I help my wife?" ([5], p.492). In Carolina, Puerto Rico, men participate actively in domestic and health issues, yet cultural values and social norms emphasised women looking after the household and family health [85]. Men and boys in Villa Francisca, Dominican Republic, assisted with hauling heavy drums of water in homes without private kitchens or bathrooms due to a locally understood and acknowledged tradition of male underemployment [111]. Unfortunately, men left them uncovered, a task associated with healthcare but not performed because health was not men’s responsibility, whilst women, who managed family health, only covered the small containers they were responsible for. This ‘gap’ between defined gender roles meant this vector control action was incomplete.

One paper attributed the gendering of women’s responsibility for dengue prevention to Indonesian culture: “…where women are supposed to do any activities within home whilst men do being responsible to any problems outside home” ([125], p.30). Nicaraguan women attributed the role division to the genders’ respective comfort: “This kind of work is better for women; men go to work in Managua, in construction, in offices. This is work inside the home. Women are more comfortable with that.” (‘Cecilia’)’; and women/men’s skills: men were too direct and lacked domestic skills, whilst women were better at time management, had social and communication skills, were more dynamic and flexible in their work ([73], p.135).

### Q2: What are the barriers to, and enablers of women's participation on an equitable basis with men in prevention practice?

The meta-synthesis identified both barriers and enablers to gender equitable prevention. Again, most categories of barriers/enablers were shared across papers (*reciprocal synthesis*) although local incarnations diverged *(refutational synthesis*). Some were identified in just one or two papers and are included to show the range. General bureaucratic and structural problems concerning how governments fund and supply prevention and control interventions are not discussed because these affected everyone.

### Barriers

#### Helplessness/lack of control

Women in several studies felt overwhelmed by the magnitude of dengue and helpless to prevent the disease through the practices and tools at hand. Whilst not strictly identified with men or men’s roles, gender inequality in prevention made women’s frustration more acute. Villa Franciscan (Dominican Republic) women reported that their efforts to create safe environments failed, and the Dominican government did not help [111]. The Klang Valley (Malaysia) housewives and others felt that prevention practices lacked benefit because neighbourhood residents made a poor effort, could not prevent the root cause of dengue if located near mosquito-saturated areas, and were uncertain about their susceptibility to illness [116]. Similarly, in Fortaleza, Brazil, women directed frustration and blame at both neighbours who failed in cleanliness and hygiene, and the government for improper street waste management [78].

#### Gendered spaces and power relations

Spaces inside the household (private) and outside (public) were regularly designated as female or male domains [73, 78, 113], which impacted the division of household labour and prevention activities, and women’s perceptions of men entering ‘female spaces’.

An older qualitative review [113] explained that across cultures and settings, instructions from formal government vector control programmes to engage in environmentally-based prevention on a domestic level could undermine women’s authority in the home. Official male patrols seeking to enter homes during the daytime to do inspections or spray chemicals when husbands and male relatives were at work could disturb women. The intrusion and forced re-organisation of domestic space for control activities could cause resentment, and violate the most private space, the master bedroom, if a male agent wanted to enter. A woman may only allow him into less private areas like the kitchen, guest bedroom or exterior to apply insecticide or inspect for mosquito larvae or triatomid bugs, leaving parts of the home unprotected. By implication, women did not hold roles as vector control agents themselves, which could have partially addressed these problems.

Male agents who disseminated health education messages to women could be resented if they implied that dengue was due to insufficient household hygienic practices. The presence of chemicals linked with asthma and allergies, or their deadly impact on domestic animals, and potential harm to pets, plants, wood or painted surfaces was also disliked. Mosquito nets or insecticide left on the walls could signify that further intrusions would occur and disempower women if the placement of these items in female-controlled space felt unfamiliar [113].

Cultural constraints and fear of crime in some countries prevent male vector control personnel from entering a home and leaving it unprotected if women were home alone without a male relative [60]. In the 1990s in Brazil, vector control agents dressed in uniforms, drove cars resembling police vehicles, and used metaphors of combat and war to describe battling with mosquitoes. These analogies could be uncomfortable for women, and off-putting as regards allowing the men to proceed [113].

Although there is evidence of projects to train and employ more female vector control agents (e.g. [25, 45]) since Winch et al.’s [113] article, according to more recent publications, the messages of the above article are still timely as women have unequal roles in dengue and MBVD prevention and control.

#### Time, poverty and money

Women were the key targets of public health interventions and campaigns of household prevention. In Villa Francisca, Dominican Republic, messages about covering water and preventing contamination were directed at the female population [111]. In Gampha District, Sri Lanka, women were chosen as the key recipients of a prevention intervention [1]. However, women were often already overwhelmed in their domestic and caring roles, more so with paid work or income generation activities [96], prevention competing with other tasks [113].

Low male participation in prevention interventions, only 20% in a community service-based intervention in West Java, Indonesia [89], burdened women with participation. In Machala, Ecuador, dengue control consumed valuable time and resources, burdening women and marginalised people [96]. Government policy could also inadvertently increase women’s burden, for example, a regional government policy to prioritise wetland protection from unsustainable human activities over urban sanitation and waste management in Celestún, Mexico, added to existing household duties [2].

#### Lack of knowledge of dengue prevention

Not fully understanding dengue transmission or how to recognise mosquito larvae impinged on successful prevention differently at individual- and community-levels. One woman in Carolina, Puerto Rico thought her backyard appeared clean, but after contracting dengue herself, detected larvae on plates underneath her flower pots [85]. Nicaragua’s *brigadistas* found it hard to convince people who scoured the city dump to implement sufficient house cleaning due to ignorance, poverty, lack of will and depression [73].

#### Devaluation of ‘women’s work’ and disempowerment

The Bandung, Indonesia study authors [89] explained that under the influence of economic rationalism, women were marginalised and patriarchal dominance endorsed as their activities of performing care and community services were not highly valued like wealth generation or increased productivity. Women predominantly implemented environmental prevention but were rarely involved in decision-making.

#### Men perceiving prevention-related administrative tasks as complex

Alongside women’s role designation inside the home, and men’s as earning income outside it, the same Bandung study appraised the reasons behind male non-participation. A key barrier was men’s perceptions that the administrative aspects of recording and reporting on community-based prevention were too complex [89].

#### Stigma and blame

Several studies reported a social stigma attached to dengue and mosquito prevention [2, 33, 96]. In Celestún, Mexico, cleaning waste had a negative public image, and in Machala, Ecuador, general perceptions of mosquito control and dengue echoed stigmas linked to uncleanliness, poverty and disease. Women in Celestún faced criticism, belittling and accusations of embarrassing themselves for being *pepenadoras* (scavengers) and removing others’ trash, also in relation to expectations that women did not ordinarily work long hours away from home. Their children were teased, and husbands and male family members often initially failed to support them or objected to women working with rubbish. Men eventually understood that recycled items represented resources and capital, when women ‘generated’ low amounts from selling discarded bottles [2]. Women were implicated in blame for poor dengue control as responsible for cleaning, and people with dengue were stigmatised as unclean and careless [96]. The Machala study’s author commented that public health campaigns about the importance of house cleaning may have strengthened social stigmas and created misperceptions that undermined the impact of formal interventions. In reverse, female local health workers and residents of Manilla regarded householders who did not inspect water containers as lazy and unconcerned by dengue [33].

### Enablers

#### Women being more health and environmentally conscious

Several papers picked up on the nuance that overall, women appeared more concerned by dengue and therefore were more invested in prevention. In Bandung, Indonesia, men conveyed more indifference to the state of the environment [89]. In Carolina, Puerto Rico, women had greater concern than men for poor waste removal, water disposal, and infrastructure contributing to transmission, and prioritised dengue due to its economic and emotional impacts and burden of disease. Men were less concerned by the underlying determinants of the disease and more concerned by an individual’s lack of understanding of health risks [85]. Women from the *Chen Kole ‘Lob* group in Celestún, Mexico, gave greater importance to environmental problems associated with dengue than men, and were unhappy about poor waste management by the government and dumping by tyre, auto repair and liquors stores, illegal dumping by the public, mosquito proliferation and absence of fumigation by government agents [2].

#### Local connections and hospitality

In Manilla, female health workers attributed local hospitality from householders, whose water containers they inspected, to the fact that one of them lived locally [33].

#### Higher levels of knowledge

Women in Celestún, Mexico, had better knowledge of dengue and its prevention than men. The *Chen Kole ‘Lob* women generated an innovative strategy for removing and recycling rubbish and raising dengue awareness. Women usually implemented fumigation and insecticides inside the house for preventing dengue regardless of whether they had personally experienced or been diagnosed with it. Only men with a previous diagnosis mentioned using mosquito repellents but not eliminating the source vector [2].

#### Common gendered experiences

Qualitative data consistently showed that teams such as the *Chen Kole ‘Lob* women and female *brigadista*s [2, 73] had bonded together to tackle dengue through shared experience of womanhood. The former were galvanised by shared, gendered experiences, concerns, and the division of labour, which made the project seem like a ‘natural’ fit. Their organisation formed away from top-down, male-focused activities [2].

#### Formal recognition of women’s abilities

The *Chen Kole ‘Lob* women’s skills and contribution to coastal waste management was acknowledged by their communities and government agencies, and endorsed their legitimacy and power as a key stakeholder in urban planning and coastal solid waste management, prompting scale up to the wider area in Yucatán [2].

#### Financial reward

*Brigadistas* were given a financial incentive for their work, and younger *brigadistas* - children or teenagers – offered scientific training, a weekly snack, a feeling of investment, and made cross-community bonds [73].

### Q3: Does women's participation result in wider community benefits, and if so, what are the health, well-being and social benefits?

Studies identified a wide range of positive benefits. These can be categorised as:

#### Individual benefits for participating women

1. knowledge - for example, that implemented and gained by *Chen Kole ‘Lob* group (Mexico) [2], and increased awareness and understanding for women’s groups participating in a dengue intervention to manage solid waste and clean up in Chennai, India [5].
2. psychosocial benefits – for example, the Chennai women’s groups experienced enhanced self-esteem, desire for involvement in community activities, and satisfaction.
3. income generation opportunities – such as those afforded to the *Chen Kole ‘Lob* women and *brigidistas* [2, 73].
4. an increase in women’s status – The *Chen Kole ‘Lob* women became known as seasoned sustainability experts in their communities, gaining more status and power [2].

#### Benefits for individuals, households and communities

1. improved prevention measures and vector reduction: the intervention in Gampha District, Sri Lanka, where women eliminated bowls, tins and bottles, reduced dengue pupal indices as a proxy for adult vector density [1]. The Chennai programme saw a definite and prolonged reduction in mosquito vectors [5].
2. psychosocial benefits, increased knowledge and decision-making - for example, women manufacturing mosquito traps from plastic bottles and solid waste for participatory community-based dengue intervention in rural Cambodia gained a feeling of community ownership of the product and its use, enhanced awareness of waste management and recycling plastic, positive change and decision-making over prevention and knowledge dissemination to schools and communities [29].
3. new opportunities, the chance to implement prevention, and community products, for example, the Cambodian intervention produced a community-owned innovation and recycling system [29].
4. an increase in men’s participation: *Chen Kole ‘Lob* began to engage men and youths to raise awareness and mobilise greater male participation in vector reduction, acting as a role model for both genders. The group is still growing and includes women and men of all ages [2].

## Discussion

This qualitative meta-synthesis of scientific and grey literature from studies published between 1991 and 2020, has shown that whole of community participation is necessary to control and prevent dengue, and as much of the burden falls on women, their increased participation and leadership is vital. I employ a gender equality ranking schema from the global aid sector, the OECD-DAC gender equality marker [77], to assess the extent to which women in this review are performing gender equitable roles in dengue prevention in LMICs. This marker draws upon Moser [72] and uses the ranks of *‘gender-sensitive’* (aims to empower women within existing structures) or *‘gender-transformative’* (aims to address the root causes of gender inequality and make significant changes to eradicate them) to evaluate an intervention or policy’s contribution. Whilst some MBVD programmes in LMICs have shown greater gender-sensitivity through recognising the importance of women’s participation, the roles that women can/do play, and collecting data on their opinions [3, 87, 101], programmes must include ‘gender transformative’ participation and leadership at all levels: in policy/strategy, implementation and delivery to address the root causes of inequity [83] in dengue prevention.

The meta-synthesis showed that women’s participation in dengue prevention and control was neither *gender sensitive* nor *transformative* with the exception of the manufacture of vector control products and sale of recycled items. In one case (*Chen Kole ‘Lob*), women were able to generate income, slightly enhanced their economic status and power, and slowly increased male participation. However, across the review at large, women’s participation reinforced traditional gender roles and divisions of labour, and increased the burden of unpaid domestic work and time poverty. Paid roles, leadership and decision-making were usually allocated to men. These findings can be complemented by those of a survey which has studied the next step forward, women’s employment in vector control, and the barriers impeding professional women’s participation in paid leadership roles in chemical vector control for malaria in Kenya, Indonesia, India and other countries [45]. The biggest barriers were “…lack of awareness of career opportunities, cultural norms, the belief that [*vector control*] is men’s work, household obligations, and lack of job security during pregnancy…”.

Going beyond Gunn et al.’s [39] systematic review that identified barriers, this meta-synthesis also identified enablers to women’s participation that played into long-standing stereotypes of ‘female qualities’ encompassing attitude, emotional intelligence, caring instincts, social and communications skills, local knowledge and understanding about health and disease (consistent with a quantitative study in Cambodia, where women had 63% higher odds of being able to name three or more dengue symptoms, likely due to their role as “care-takers”) ([52], p.11), objection to gender inequity, and environmental interests. The identified qualities show some consistency with a qualitative MBVDs study [32] where formal vector control stakeholders in Kenya and Indonesia agreed that women’s daily presence in, and knowledge of households and surroundings, was greater than men’s. Women were found to be better communicators, more trustworthy and pro-active, better at networking, and have stronger networks for implementing protective measures ([116], see also [18]). However, the identification of women’s qualities alone is neither *gender sensitive* nor *transformative* without real opportunities to use them in leadership and decision-making as the means to greater empowerment 

## Recommendations

Whilst it is beyond the scope of this paper to present a complete action manifesto for achieving gender *sensitive* or *transformative* gender role changes and women’s empowerment in dengue prevention and control, I offer some basic recommendations. A multi-pronged, multi-level approach is required. Greater targeted advocacy will be required from influential agents such as government health bodies, especially vector borne disease control units writing gender equity into the implementation of their policies and strategies, with supporting efforts from civil society, and commercial stakeholders (e.g. those manufacturing disease prevention and control products). This advocacy should be supported by public awareness campaigns delivered through electronic and social media including audiovisual and visual advertising and using the arts (e.g. music, drama, storytelling, film, visual art) to engage populations, especially the illiterate.

Women should be given leadership roles in community-based vector control programmes, small businesses and paid roles in large-scale programme implementation. There are huge implications for future dengue control. Some inspiration can be taken from a very large-scale official malaria prevention initiative funded by international donor, the United States Aid Agency (USAID). *The U.S. President’s Malaria Initiative (PMI) Africa Indoor Residual Spraying (AIRS) Project,* implemented a number of prevention methods such as indoor spraying of insecticides to kill malaria mosquito vectors in 19 African countries, and attempted to increase the number of women employed in paid positions and supervisory roles in official spraying patrols. The barriers to participation were analysed and then gender-guided policies implemented in Benin, Ethiopia, Ghana, Mali, Madagascar, Mozambique, Rwanda, Senegal, Zambia, and Zimbabwe starting in 2015. The percentage of women employed in in supervisory roles rose from 17% (2012) to 46% (2015) [25].

However, rather than remaining dependent on foreign donor aid programmes, effective dengue prevention necessitates that individual country governments put efforts into running gender equitable dengue vector control programmes that prioritise environmental management that is cheap to implement, and which could become financially self-sustaining. Womens’ entrepreneurship is an obvious target for this. Women need opportunities and support to generate income, and mentoring and support to develop their own leadership styles. The authors of the aforementioned survey of barriers facing women to entering chemical vector control roles to combat malaria highlighted that one of the most effective strategies for increasing women’s participation and leadership was the availability of micro-finance to start their own vector control business ([45]; see also [80] for this perspective within all health promotion). Women programme leads and supervisors of dengue control could organise and lead patrols of people removing water-holding vessels such as discarded tyres and empty containers from the streets, carry out street cleaning, recycle viable items, and manufacture water tank and water vessel covers and other prevention devices to sell to the authorities, businesses, communities and households. They could offer peer support to, and train emerging supervisors, and other women and men in the full range of prevention tasks.

Programmes should address women and their roles separately from mixed gender programmes in the first instance so as to train them, build confidence and empower them into leadership and decision-making roles separately from men. The ultimate aim is to create a level playing field for female leadership whilst encouraging greater male participation across the spectrum of tasks required in dengue prevention. But in the present, women must be allowed to grow their skills in leading roles in the first instance in comfortable, safe spaces where they can question established norms and roles, and creatively explore and grow styles and modes of leadership and decision-making on their own terms. They need the full backing and support of organisations which are accepted by men in their communities, and which are able to open and hold dialogues with male community leaders and representatives to get them on side to influence men (and women) in their communities.

Women’s leadership styles may differ from traditional patriarchal leadership styles, but may result in more successful outcomes. There are many different types of formal and informal agencies and organisations, both top-down formal, and bottom-up grass-roots groups, that could support and implement this work. These include: international organisations, donor agencies, NGOs, civil society organisations (CSOs), participatory research programmes based at universities and independent research organisations, unions, faith-linked groups and informal women’s collectives and coalitions. Outreach programmes can also be delivered in ‘settings’ such as “schools, villages, workplaces, churches, radio talk-back shows …” ([44], p.21). Researchers investigating the role of gender in Zika prevention behaviours in the Dominican Republic raised an essential issue for reducing the burden of Zika and other similar mosquito-borne diseases (i.e. dengue): that gender must be integrated into prevention programmes with due sensitivity for the local cultural context and without taking advantage of existing gender roles [40].

Local women should be consulted as to which organisations have a positive and appropriate influence on women’s empowerment before dismissing certain bodies that may not appear as a likely choice, for example, women’s church-linked groups in the Pacific Islands Countries and Territories (PICT) (Melanisia, Micronesia, and Polynesia). In the Solomon Islands and Melanesia, which are patriarchal, conservative societies, these groups are socio-culturally important. Despite outward religious conservatism, they are described as a hotbed for women’s empowerment via opportunities provided for networking with external organisations, building solidarity, informal education and gaining certain types of leadership and decision-making experience in women-friendly contexts [92]. Church-linked groups and churches themselves hosted, for examples, sexual health awareness training for female community leaders and village health workers although with respect to local social and cultural features such as religion and gender [74]. Other women’s health initiatives facilitated include mammogram screenings [49], health literacy training and discussion and action against domestic violence [92]. Similar opportunities could be facilitated through parallel groups in other societies to support the normalisation of women’s leadership roles and decision-making powers in dengue prevention.

Funding a squad of dengue prevention champions in urban areas, as was observed in this review with the *brigadistas* of Cuidad Sandino, Nicaragua [73] and building on the existing skills of women village health workers in rural areas by selecting and funding them to attend female leadership training could be a productive strategy. There is evidence of village health workers playing major roles in the prevention of other vector borne diseases, for example, *The Blue Nile Health Project* that ran with a gender emphasis for 10 years in Central Sudan primarily for the control of water-associated diseases. Female health instructors or *murshidat*, who were preferred in the matrilineal conservative Muslim society, and female village health committee members played a major part in motivating, organisation and providing health education to local communities ahead of campaigns promoting “environmental sanitation and vector control…” ([86], p.1422). Yet they had less involvement in the environmental tasks of drying mosquito breeding sites and spraying insecticides. Dengue control programmes should increase women’s roles to include environmental management tasks and firmly transform urban or village health workers into project leaders by building their confidence and knowledge including about their rights, providing micro-credit to develop their own or cooperative businesses, ensuring access to facilities and essential services, and further developing their professional skills [80].

The role of children as active agents in prompting and protecting their own health is acknowledge in literature discussing the benefits of treating the family unit as a locus for health promotion and disease prevention (e.g. [20, 69]). Children should develop greater awareness of dengue and its prevention and control methods through schools and community education and family disease prevention programmes. They should be shown examples of women’s leadership and empowerment in disease prevention, which they can usefully reinforce to their mothers and families when discussing what they learnt about dengue. Children as well as adults also have networks of their peers through which to disseminate information and learning. However, none of the papers reviewed here gave the impression that community-based women needed persuading of the importance of implementing vector control and dengue prevention. They were overburdened with it at household level but mainly lacked paid roles and leadership opportunities.

The risk of male backlash, including GBV, against newly-empowered women in the global south, must be identified and mitigated using norm shifting techniques, such as role-play exercises where women and men exchange gendered roles and act out scenarios where traditional norms are gently challenged. Male community leaders and representatives need to be persuaded to encourage males to get involved in exploratory socio-cultural norm shifting to eradicate violence against women and girls and pave the way for male receptivity to female leadership over time. Another option is family-based counselling [124] which identifies and challenges unhelpful norms whilst exploring the family benefits of women’s entrepreneurship and income generation, for example, relieving pressure and stress from men as sole economic providers. Women must have societal freedom from discrimination and violence as well as access to economic resources and social inclusion to generate and maintain their health and well-being, which also benefits men [80].

These changes could mean more efficient and cost-effective dengue prevention and control, reduced disease rates and costs of medical care and treatment for government services and sectors. Dengue prevention and control policies and strategies should be *gender transformative* and address the root causes of gender inequity through being accompanied by deeper structural and legal changes to eradicate it altogether. The findings endorse additional existing recommendations [60, 85, 96, 111] including encouraging all household members to engage in prevention interventions and family healthcare, broadening men’s roles, targeted public health information and education campaigns to reduce stigmas and emphasise public knowledge about dengue and gender equitable prevention, or even separate messages for women and men.

Further research should be conducted on men’s roles, which are poorly detailed. In just one study [85], the boundary between gendered roles was more blurred, an issue that deserves further investigation given that some prevention tasks remained unallocated to women or men. The gendered dynamics of informal and formal prevention should be studied to unpack men’s understandings of the disease and vector control; both well-documented and undocumented actions they performed. The polarisation of female/male roles and responsibilities needs to be understood to see where there is malleability to blur this boundary, involve more men, and engage women’s strengths in leadership and decision-making. It is long established that when health promotion campaigns address whole families and the relationships between women and men of all ages, health programmes can be substantially improved [80]. For example, in Ghana, information about the vitality of immunisation was given to both mothers and fathers, which encouraged men to assume more responsibility for children’s health and led to an increased surge of vaccine take-up and preceding immunisations [14]. Similar achievements were seen in a reproductive health outreach programme in Lao PDR [30]. If this approach was adopted in dengue and MBVD prevention and control alongside norm shifting work to garner the acceptability of women’s leadership and decision-making powers given their strengths around dengue prevention, men could take up more responsibility for everyday tasks and women leaders of community-based programmes could emerge.

## Strengths and limitations

This review is the first known qualitative meta-synthesis into gender equity in dengue prevention and control, and as such, yields a variety of insights into women’s participation, barriers, enablers and benefits. It has been conducted with methodological rigour given the broad range of databases and focus on health outcomes, and the extensive grey literature search. This was important as some dengue prevention interventions are owned, implemented and reported on by government agencies, UN agencies, and non-governmental organisations who may not publish their activities in peer-reviewed journals. It cannot claim to be exhaustive as the purpose is interpretation not quantification. The quality appraisal process added to the methodological rigour by using a thematically appropriate tool, the JBI Critical Appraisal Checklist for Qualitative Research, which was designed for health sector research, chosen over the commonly used Critical Skills Appraisal Programme (CASP) [21] Qualitative Research Checklist. This meta-synthesis complements the scale provided by statistical data with its depth of insights, which can be supplemented. Another strength is its methodological contribution to a greater understanding of the role of gender in dengue control, and in successful prevention programme implementation.

There are several limitations, firstly having a second person or a group that would double or randomly check the process would have enhanced the quality although this paper has received rigorous feedback from colleagues at the University of New South Wales (Dr Anita Heywood and Dr. Husna Razee) and additionally from colleagues in Tanzania (Dr Opportuna Kweka, University of Dar Es Salaam, and Dr. Leonard Mboera, Sokoine University of Agriculture). Secondly, the generalisability of findings from specific socio-cultural contexts to other locations, as noted by Gunn et al. [39]. Their study and this report significant variations between gendered norms, women’s status and rights in different geographic regions. Another limitation is the geographic publication bias towards studies from Latin America and the Caribbean and South East Asia. Dengue is a comparative recent phenomenon in Africa where scholarship has predominantly focused on malaria. This may also be owed to funders prioritising malaria, the better-known and previously more widely spread disease, but with some regional successes in malaria control, dengue has started to command more government awareness. This may be followed by increased dengue scholarship from African scholars.

## Conclusions

The maximum participation of as many people as possible is urgently needed in dengue-affected communities in future with climate change predictions, yet without women’s full participation and leadership, there is a chronic waste of important human resources. The continuation of unhelpful patriarchal norms and power structures, in which dengue interventions are embedded, have so far failed to curb the disease. The strengths and qualities exhibited by women such as specialist health and medical knowledge, and listening and social skills, networking and influencing, may make them more effective leaders in steering communities through disease prevention. The clear benefits of group participation, including sense of control, coherence and personal identity [10], agency, efficacy [8], empowerment [22, 38, 56, 57] ad aptive capacity [7], enhanced personal and coping skills [56], social and emotional well-being [10, 55] and resilience would strengthen communities’ abilities to address broader health, poverty, and climate resilience challenges [7, 27, 39, 57, 90, 120]. Women and their families and communities are likely to experience significant psychosocial benefits from individual and group participation.

Additionally, women’s awareness and skills in everyday environmental management for disease prevention but also for sanitation and hygiene practices, ecosystem preservation, resource management, climate mitigation and others are critical at a time when pressures on natural resources, ecosystem destruction and climate crises are paramount. Dengue prevention and control is just one example of the public health benefits afforded by the greater participation and recognition of women in communities and as an integral part of the workforce.

## Data Availability

Not applicable.

## Appendix

Tables 3, 4, 5 and 6.

**Table 3:** Key words used in the search strategy

Initial Scan	Dengue AND (gender or women or female) AND (prevention and/or control)
Additional keywords for initial broad searches:	(intervention or program/me or practice/s) AND (communities) AND/OR (formal or informal) AND/OR (participation or engagement or consultation or involvement or deliberative) OR (policy or health or healthcare or system or government or civil society) AND (qualitative research) AND (vector or mosquito) AND (urban or environment/al) AND (upgrading or development or planning or design or management or manipulation or modification) AND (global south or country or nation/s) AND (Africa or Asia or South East or Latin America or Pacific)
And strings of^a^:	(vector-borne disease or communicable disease or neglected tropical disease or infectious disease) AND (protect/ion or measures) AND (girls or men or boys or male or transgender or non-binary) AND/OR (equality or equity or inclusion/include/d or exclusion/exclude/d or change) AND (integrated or multi-sectoral/multisectoral or social or preparedness) AND/OR (emergency response or outbreak or epidemic or endemic or pandemic) AND/OR (pathogen or sickness or illness or wellbeing or well-being) AND/OR (international and/or development or governance or health systems strengthening or World Health Organization or health sector or global health policy or health policy or Ministry of Health or plan or strategy) AND/OR (ecosystem or climate change or pollution) AND/OR (clinical or risk or causal and/or pathway or determinants or epidemiology) AND/OR (people or family or households or tribe or village) AND/OR (socio-economic or jobs or business) AND/OR (empowerment) AND/OR (hierarchy or structure/ral) AND/OR (hospital or clinical or facility or medication) AND/OR (water or waste or sanitation or fogging or net or spray/ing or container or upturned or pipe or bed and/or net or WASH or chemical or land or access) AND (vulnerable or marginalised or discrimination or minority) AND/OR (monitoring and/or evaluation)

^a^Descriptive data on dengue scenarios are often buried in papers centred on a variety of diseases and do not come up in key word searches using the initial obvious key words, so the broader keywords in row three were employed

**Table 4:** Number of titles returned from refined search

Name of database or search engine	No of titles returned using combinations of the terms: (dengue) AND (gender or women or female) AND (prevention and/or control)
University of Glasgow Library Catalogue	3025 (with gender only) (first 100 titles); 6191 (with women only); 11,492 (with female only); (first 100 titles)
UNSW Library Catalogue	4033 (with gender only) (first 100 titles); 11,441 (with women only) (first 100 titles); 13,854 (with female only) (first 100 titles)
Google Scholar	24,900 (with gender only) (first 100 titles); 38,400 (with women only) (first 100 titles); 42,400 (with female only) (first 100 titles)
Pub Med	160 (with gender only)’ 158 (with women only); (with female only) 1805 (first 100 titles)
Medline	2 (with gender only); 0 (with women only), but based on keywords 4902 (first 100 titles); (with female only) 2
PsychINFO	94 (with gender only); 93 (with women only); (with female only) 127 (first 100 titles)
ProQuest	5321 (with gender only) (first 100 titles); 8243 (with women only) (first 100 titles); 9854 (with female only) (first 100 titles)
Scopus	43 (with gender only); 95 (with women only); 791 (with female only) (first 100 titles)
EMBASE	5465 (with gender only); 3138 (with women only) (first 100 titles); 4725 (with female only) (first 100 titles)
Web of Science	77 (with gender only); 247 (with women only) (first 100 titles); 2170 (with female only) (first 100 titles)
EBSCOhost-Medline	2 (with gender only); 4775 (with women only) (first 100 titles); 2 (with female only)
Psychology & Behavioural Sciences Collection	51 (with gender only); 54 (with women only); 59 (with female only)

**Table 5:** Grey Literature Search (reproduced from Gunn et al. 2017, p.4)

Organisation	Website	Relevant Papers
African Development Bank	http://www.afdb.org/en/	NO
Asian Development Bank	http://www.adb.org	1 (found through research database)
Australian Agency for International Development	http://www.dfat.gov.au/aid/	NO
Bayer	http://www.bayer.com	NO
CARE International	http://www.care-international.org	NO
CARE US	http://www.care.org	NO
CropLife International	http://www.croplife.org	NO
Department for International Development	http://www.gov.uk/government/organisations/department-forinternational-development	NO
European Centre for Disease Prevention and Control	http://www.ecdc.europa.eu	NO
Fiocruz (Brazil)	http://portal.fiocruz.br/en	NO
Gates Foundation	http://www.gatesfoundation.org	NO
Innovative Vector Control Consortium	http://www.ivcc.com	NO
Inter-American Development Bank	http://www.iadp.org	NO
International Initiative for Impact Evaluation	http://www.3iempact.org/	NO
International Institute for Environment and Development	http://www.iied.org	NO
International Livestock Research Institute	http://www.ilri.org	NO
International Monetary Fund	http://www.imf.org	NO
Oxfam	http://www.oxfam.org	NO
President’s Malaria Initiative	http://www.pmi.gov	NO
Roll Back Malaria	http://www.rollbackmalaria.org	NO
The Global Fund	http://www.theglobalfund.org	NO
United Nations Development Program	http://www.undp.org	NO
United Nations http://www.un.org/en/	United States Centers for Disease Control and Prevention http://www.cdc.gov	NO
United States Agency for International Development	http://www.usaid.gov	NO
Vector Control Research Centre	http://www.vcrc.res.in	Page Not Available
Vestergaard Frandsen	http://www.vestergaard.com	NO
Wellcome Trust	http://www.wellcome.ac.uk	NO
Women Organizing for Change in Agriculture and Natural Resource Management	http://www.wocan.org	1 (Published by UNEP)
World Agroforestry Centre	http://www.worldagroforestry.org	NO
World Bank	http://www.worldbank.org	1
WHO	http://www.who.int	1
World Vision	http://www.worldvision.org	NO
**Additional organisations added by author**		
UN Women	www.unwomen.org/en	NO
Malaria Consortium	https://www.malariaconsortium.org/	NO
United Nations Environment Programme (UNEP) Gender and Water Alliance	https://www.unep.org/	1 (found through research database)

**Table 6:** Summary of literature search results

No	Citation	Reason for exclusion
1	Bassoa,C., Garcıa da Rosa, E., Romeroc, S., et al. (2015) *Transactions of the Royal Society of Tropical Medicine and Hygiene* 109, pp. 134–142	A
2	World Bank (2008) *Environmental health and child survival: epidemiology, economics, experiences*. II. Series: Environment and development. Washington, D.C.: World Bank.	A
3	Alobuia, W.M., Missikpode, C., Aung, M., et al. (2015) *Annals of Global Health*. 81(5), pp.654–63.	A
4	Chiaravalloti Neto, F., de Moraes, M.S., et al. (1998) *Cadernos de Saúde Pública*. 14 Suppl 2, pp.101–9. Portuguese.	B
5	Crabtree, S.A., Wong, C.M., Mas’ud, F (2001). *Human Organization* 60 (3) p.281	E
6	Chiaravallot, N.F., Baglini, V., Cesarino, Favaro, E.A, et al. (2007) *Cadernos de Saúde Pública*. 23 (7), pp.1656–64. Portuguese.	B
7	Nava-Aguilera, E., Morales-Pérez, A., Balanzar-Martínez, A., et al. (201). *BMC Public Health* 30 (17) (Suppl 1), p.435.	A
8	Hiaravalloti, V.B., Morais, M.S., Chiaravalloti, N.F., et al. (2002) *Cadernos de Saúde Pública* 18 (5), pp.1321–9. Portuguese.	B
9	Lloyd, L.S., Winch, P., Ortega-Canto, J., et al. (1992) *American Journal of Tropical Medicine and Hygiene* 46 (6), pp.635–42.	A
10	García-Betancourt, T., Higuera-Mendieta, D.R., González-Uribe, C., et al. (2015) *PLoS ONE* 10 (6), p.e0129054.	D
11	Elsinga, J., Schmidt, M., Lizarazo, E.F., et al. (2018) *American Journal of Tropical Medicine and Hygiene* 99 (1), pp./195–203.	A
12	Arenas-Monreal, L., Piña-Pozas, M., Gómez-Dantés, H (2015) *Salud Publica de Mexico* 57(1), pp.66–75. Spanish.	Article in Spanish
13	Andersson, N., Nava-Aguilera, E., Arosteguí, J.(2015) *British Medical Journal* 351, pp.h3267.	A
14	Paul, M.R.M (2006) *A study to assess the knowledge and practice of women regarding prevention of Dengue fever in Singasandra PHC area, Bangalore South with a view to develop an information booklet.* Karnataka, Bangalore, India: Masters Dissertation submitted to the Rajiv Ghandi University of Health Sciences.	A
15	Sommerfeld. J and Kroeger, A (2015) *Transactions of the Royal Society of Tropical Medicine and Hygiene* 109 (2), pp.85–8.	A
16	Andersson, N (2017) *BMC Public Health* 17 (Suppl 1), p.397.	A
17	Sulistyawati, S., Dwi Astuti, F., Rahmah Umniyati, S., et al. (2019) *International Journal of Environmental Research and Public Health* 16 (6),p.1013.	A
18	Kusuma,Y.S., Burman, D., Kumari, R., et al. (2019) *Global Health Promotion* 26 (1), pp.50–59.	A
19	Kendall, C., Hudelson, P., Leontsini, E., et al. (1991) *Medical Anthropology Quarterly,* Sep.,1991, New Series, 5(3), pp. 257–268. Contemporary Issues of Anthropology in International Health.	E
20	Van Benthem, B.H., Khantikul, N., Panart, K., et al. (2002) *Tropical Medicine and International Health* 7 (11), pp.993–1000.	A
21	Therawiwat, M., Fungladda, W., Kaewkungwal, J., et al. (2005) *Southeast Asian Journal of Trop Medicine and Public Health* 36(6), pp.1439–49.	A
22	Halton, K., Sarna, M., Barnett, A., et al. (2013) *Jbi Database of Systematic Reviews and Implementation Reports* 11(2), pp.1–235.	Quantitative studies only
23	Cockcroft, A (2017) *BMC Public Health* 17 (Suppl 1), p.409.	A
24	Rakhmani, A.N., Limpanont, Y., Kaewkungwal, J., et al. (2018) *BMC Public Health* 18, p.619	A
25	Andersson, N., Beauchamp, M., Nava-Aguilera, E., et al. (2017). *BMC Public Health*, 17 (Suppl 1), p.408.	A
26	Pengvanich, V (2011) *Journal of the Medical Association of Thailand*. 94 (2), pp.235–41.	A
27	Podder, D., Paul, B., Dasgupta, A., et al. (2019) *Indian Journal of Public Health* 63, pp.178–85	A
28	Sunil, A., Gnanadurai, A., Anto, T (2017) *Asian Journal of Nursing Education and Research* 7(10), pp. 35–42.	A
29	Kholedi, A.A., Balubaid, O., Milaat, W., et al. (2012) *East Mediterranean Health Journal* 18(1), pp.15–23.	A
30	Firdous, J., Mohamed, A., Al Amin, M., et al. (2017) *Malaysia Journal of Applied Pharmaceutical Science* 7 (08), pp.99–103	A
31	Faridah, L., Nuriyah, E., Ekawardhani, S., et al. (2019) *Juran Endurance: Kajian Ilmiah Problema Kesehatan* 4(1), pp.210–219.	A
32	World Health Organization (WHO) and the Special Programme for Researchand Training in Tropical Diseases (TDR) (2009) *Dengue: guidelines for diagnosis, treatment, prevention and control -- New edition*. Geneva: World Health Organisation.	A
33	Soedarmo, S.P. (1993) *Tropical Medicine* 35 (4), pp.315–324.	A
34	Shafique, M., Lopes, S., Doum, D., et al. (2019) *PLoS Neglected Tropical Diseases* 13(11), p.e0007907	C
35	Khun, S., Manderson, L (2007) *PLoS Neglected Tropical Diseases* 1(3), p.e143.	Women discussed only as passive recipients of health education for prevention.
36	Frank, A.L., Beales, E.R., de Wildt, G., et al. (2017) *PLoS Neglected Tropical Diseases* 11(9), p.e0005755.	C
37	Zuhriyah, L., Mayashinta, D.K., Kurnianingsih, N., et al. (2019) *Journal of Public Health in Africa* 10(s1):1208.	No gender analysis in qualitative data
38	Dickin, S.K., Schuster-Wallace, C.J., Elliott, S.J. (2014 *Applied Geography* 46, p.71e79	A
39	Daude, E., Mazumdar, S., Solanki, V (2017) *PLoS ONE* 12(2), p.e0171543.	A
40	Idalí Torres, M (1997) *Human Organization* 56 (1), pp.19–27.	D
41	Boischio, A., Sánchez, A., Orosz, Z., et al. (2009) *Cad. Saúde Pública*, Rio de Janeiro, 25 Sup 1: pp.S149-S154.	A
42	Tapia-Conyer, R. Méndez-Galván, J., and Burciaga-Zúñiga, P (2012) *Paediatrics and International Child Health*, 32: sup1, pp.10–13,	A
43	Harapan, H., Anwar, S., Bustaman, A., et al. (2016) *Asian Pacific Journal of Tropical Medicine* 9 (11), pp.1115–1122.	A
44	Kyu, H.H., Thu, M., and Van der Putten, M (2005). *Assumption University Journal of Technology* 9(2), pp.99–105	A
45	Wong, L.P., Shakir, S.M.M., Atefi, N., et al. (2015) *PLoS ONE* 10(4), p.e0122890.	A

Decisions to Exclude

A – No qualitative data on women’s role in prevention

B – Article in Portuguese

C - Insufficient gender analysis in qualitative data

D - Removed from meta-synthesis during sensitivity analysis

E – No gender analysis
